# The external validity of a novel contract‐relax stretching technique on knee flexor range of motion

**DOI:** 10.1111/sms.13554

**Published:** 2019-10-03

**Authors:** Anthony D. Kay, Joshua Dixon, Liam D. Bligh, Anthony J. Blazevich

**Affiliations:** ^1^ Centre for Physical Activity and Life Sciences University of Northampton Northampton UK; ^2^ Centre for Exercise and Sports Science Research (CESSR) School of Medical and Health Sciences Edith Cowan University Joondalup WA Australia

**Keywords:** ecological validity, flexibility, muscle stretching basic science to clinical practice, PNF

## Abstract

**Introduction:**

Compromised joint range of motion (ROM) can negatively affect the capacity to perform activities of daily living in clinical populations. Recently, similar improvements in dorsiflexion ROM were reported following dynamometry‐based contract‐relax (CR) stretching and modified CR stretching technique (stretch‐return‐contract [SRC]) where the contraction phase was performed “off stretch.” As neither the impact of SRC on other muscle groups nor the ecological validity of SRC performed in an applied environment has been tested, the acute effects of both techniques in dynamometry‐ (CR_dyna_ and SRC_dyna_) and field‐based (CR_field_ and SRC_field_) environments were compared with the hamstring muscle group.

**Methods:**

Seventeen participants performed each of the four stretching conditions on separate days in a randomized order. Before and after the stretches, knee extension ROM and passive knee flexor moment were recorded on an isokinetic dynamometer.

**Results:**

Significant (*P* < .01) increases in knee extension ROM (4.6‐5.2°) and elastic potential energy storage (12.0%‐23.6%) and decreases in the slope of the passive moment‐angle relation (8.9%‐12.2%) occurred in all conditions. Significant increases in peak passive joint moment were observed after field‐ (14.3%‐14.8%) but not dynamometry‐based (4.6%‐6.6%) stretches. No difference (*P* > .05) in any measure was found between conditions.

**Conclusions:**

These data confirm the acute efficacy of the SRC technique in the hamstring muscle group and demonstrate its ecological validity in an applied environment in healthy participants. As the field‐based SRC technique was performed without partner assistance, when compared with classical PNF it represents an equally effective and practical stretching paradigm to support athletic and clinical exercise prescription.

## 1 INTRODUCTION

Lower‐limb joint ranges of motion (ROM) and passive resistance to stretch during joint rotations (indicative of tissue stiffness) are important functional parameters that influence physical function and muscle strain injury risk^1^ and are compromised in a range of conditions including diabetes,^2^, ^3^ cerebral palsy,^4^ stroke,^5^ aging,^6^ and arthritis.^7^ While static muscle stretching is both easily applied and commonly used in both clinical and athletic environments to increase ROM, proprioceptive neuromuscular facilitation (PNF) stretching techniques are often reported as being more effective for promoting both acute and chronic improvements.^8^, ^9^, ^10^ One common method of PNF stretching is the contract‐relax (CR) technique, where repeated cycles of static stretching and intense, often maximal, isometric contractions are performed in a fully stretched position. While this method of stretching has been found to be successful in substantially improving ROM,^11^ drawbacks can include the requirement for an assisting partner and the contractions being performed at long muscle lengths, which are often painful and result in greater symptoms of muscle damage (ie, reduced strength and ROM, increased tenderness).^12^, ^13^ The necessity to stretch the musculature fully prior to initiating these contractions during PNF techniques may be problematic for any population to perform that exhibits muscular hypertonicity, such as spasticity or contracture, where ROM is often compromised and muscles cannot be stretched to their full length.^14^ Furthermore, while the use of bands or a towel may enable PNF stretches to be performed alone in some muscle groups (eg, hamstrings), a partner or clinician is often required thus preventing outpatients from using PNF following clinical discharge and may, in some part, also explain why it is not more commonly used in athletic environments. Therefore, while CR stretching is highly effective and used in clinical populations to achieve rapid increases in ROM, important limitations restrict its more general use.

Similar acute increases in dorsiflexion ROM have been reported following maximal isometric contractions performed “off stretch” (ie, at shorter muscle lengths) to those observed following static stretching.^15^ Importantly, the increase in ROM following isometric contractions was achieved without any muscle stretching being imposed. This finding prompted the development, and subsequent assessment, of a modified CR technique (stretch‐return‐contract [SRC]) in which the contraction phase was performed “off stretch” (ie, at shortened muscle length) between successive passive static stretching cycles.^16^ Using this technique, identical acute enhancement of dorsiflexion ROM and changes in muscle‐tendon mechanics were observed. However, both the training and the testing were performed on an isokinetic dynamometer, so it not known whether the method is effective when performed by an individual without dynamometer assistance. Therefore, the ecological validity of the modified technique remains unknown.

Given the limitations described above, the purpose of the present study was to test the feasibility and effectiveness of the modified (SRC) technique in practice in order to determine whether it can be successfully performed without a partner or isokinetic dynamometer assistance and in muscle groups other than the plantar flexors. As this was a proof of concept study, it was decided to test these effects in a healthy population before investigations were initiated in clinical populations. Therefore, the aims of the present study were to examine and compare the acute effects of two stretching methods (CR vs SRC) in two environments (laboratory‐ vs field‐based) on knee extension ROM, maximal isometric knee flexor moment, peak passive joint moment at full volitional ROM (stretch tolerance), the slope of the passive moment curve (indicative of whole muscle‐tendon complex [MTC] stiffness), the area under the passive moment curve (indicative of elastic potential energy storage), and muscle EMG activity during stretches. We tested the hypothesis that CR and SRC stretching techniques performed in laboratory‐ and field‐based environments would produce similar changes in all measures.

## 2 MATERIALS AND METHODS

### 2.1 Participants

Seventeen recreationally active participants that were not habitually engaged with intense flexibility or resistance training (7 women, 10 men; mean (SD) age = 27.7 (9.2) year, height = 1.7 (0.1) m, mass = 73.4 (17.9) kg) with no recent history of lower‐limb injury volunteered for the study after completing a pre‐test medical questionnaire and providing written and informed consent. The participants were asked to avoid any flexibility training, intense exercise, and stimulant use for 48 hours prior to testing. Ethical approval was granted by the Faculty of Health and Society's Ethics Committee at the University of Northampton with the study completed in accordance with the Declaration of Helsinki.

### 2.2 Protocol

#### 2.2.1 Participant positioning

**Figure 1:**
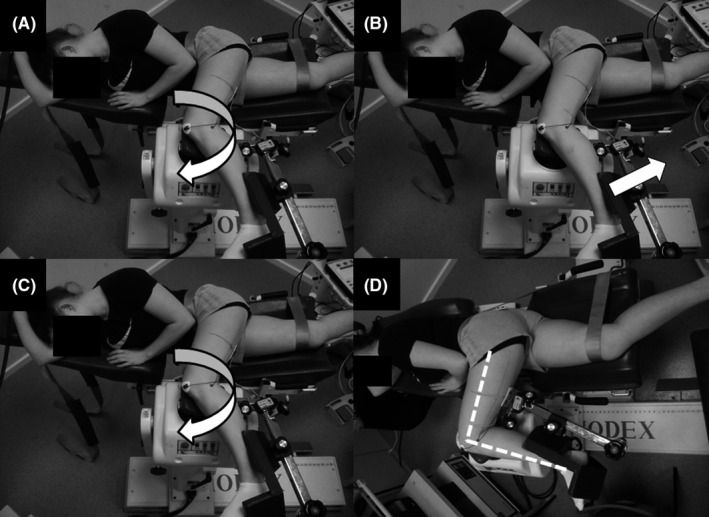
Participant positioning on the dynamometer during laboratory‐based contract‐relax (CR) and modified CR (stretch‐return‐contract [SRC]) stretching protocols. During CR (A) and SRC (C), the participant's left knee was passively rotated to the point of discomfort. The contraction phases during CR (B) and SRC (D) were performed at either full stretch (CR) or 90° flexion (SRC), respectively

The participants were familiarized with the experimental testing and stretching protocols one week prior to data collection and visited the laboratory on four further occasions under experimental conditions, performed in a randomized order with each trial separated by 48 hours. During the experimental trials, the participants performed a 5‐minute warm‐up on a Monark cycle at 60 rpm with a 1‐kg resistance load. The participants were then positioned in the fully reclined chair of an isokinetic dynamometer (Biodex System 3 Pro, IPRS) laying on their right side. The right leg was placed in the anatomical position (0°) at the hip and knee with non‐elastic strappings across the hips and right thigh to minimize pelvic rotation and anterior pelvic tilt, respectively. The left shank was strapped in the dynamometer's leg attachment with the left hip flexed to 120° and knee flexed to 90°, with the medial femoral epicondyle aligned over the center of rotation of the dynamometer (see Figure 1D). The position and strappings were adopted from methods employed in previous studies examining knee flexor moment and stiffness^17^, ^18^ to ensure that gravitational effects on the shank could not influence passive moment during ROM trials.

#### 2.2.2 Isometric knee flexor moment

During the active trials, the participants performed two warm‐up submaximal isometric knee flexor contractions at 50% and 75% of perceived maximum voluntary isometric contraction (MVC) on the left limb followed by two ramped MVCs, with MVC reached ~3 seconds after contraction initiation and held for 2 seconds (60 seconds rest between contractions). Joint moment and angle data were directed from the dynamometer to a high‐level transducer (model HLT100C, Biopac) before analog‐to‐digital conversion at a 2000‐Hz sampling rate (model MP150 Data Acquisition, Biopac). The data were directed to a personal computer running AcqKnowledge software (v4.1, Biopac) and filtered using a zero lag, 6‐Hz Butterworth low‐pass filter. The greater of the two isometric MVCs was used as a measure of maximal isometric joint moment, where >5% difference in moment occurred a third contraction was performed.

#### 2.2.3 Muscle activity

Uniform/comparable activation patterns during stretching have been reported within the hamstring muscles^18^; thus, skin‐mounted bipolar double differential active electrodes (model MP‐2A, Linton) were only placed over the semitendinosus muscle with a reference (ground) electrode over the tibia with raw EMG data constantly monitored during the active (MVC) and passive (ROM) trials. Semitendinosus EMG data collected during the contractions were amplified (gain = 300, input impedance = 10 GΩ, common‐mode rejection ratio ≥100 dB at 65 Hz) and directed to a high‐level transducer (model HLT100C, Biopac) before analog‐to‐digital conversion at a 2000‐Hz sampling rate (model MP150 Data Acquisition, Biopac). The data were stored on a personal computer running AcqKnowledge software (v4.1, Biopac) and processed using a 20‐ to 500‐Hz band‐pass filter and converted to root‐mean‐squared EMG with a moving 250‐ms averaging window. The EMG data were then normalized as a percentage of the mean of the peak EMG amplitudes obtained in the two pre‐stretching MVC trials. The normalized EMG amplitude (%MVC) was used as a measure of neuromuscular activity, which was then quantified within a 250‐ms epoch at peak joint moment during the greater of the two MVCs performed before and after the interventions.

#### 2.2.4 Range of motion, passive moment and reflexive EMG

Two minutes later the participants performed three passive knee extension trials initiated from 90° knee flexion at 0.087 rad/s (5°/s) until the participants volitionally terminated the rotation with a hand‐held stop button at the point of discomfort, a stretch intensity commonly used in ROM studies.^19^, ^20^ The passive trials enabled ROM, peak passive moment at full ROM (ie, stretch tolerance), elastic potential energy storage (ie, area under the curve), the slope of the passive moment curve (indicative of MTC [joint] stiffness), and reflexive EMG to be calculated. Peak passive moment (Nm) and EMG (%MVC) were measured within a 250‐ms epoch at full volitional ROM from the third passive ROM trial to ensure thixotropic properties of skeletal muscle did not influence the joint moment data.^21^ The slope of the passive moment curve (Nm/°) was calculated as the change in knee flexor moment through the final 10° of knee extension (ie, in the linear portion of the passive moment curve) in the pre‐stretching trials. Identical joint angles were used in the post‐stretching trials enabling the same region of the passive moment curve to be analyzed, which ensured any changes in stiffness data were a likely consequence of changes in MTC stiffness rather than examining a different region of the curvilinear passive moment curve.^15^ Elastic potential energy storage (J) was calculated as the area under the passive moment curve from 90° flexion (ie, starting position) through to full ROM.

#### 2.2.5 Stretching interventions

During the laboratory‐based conditions (ie, stretches performed on the dynamometer), the knee was passively extended from a starting position of 90° of flexion at 5°/s until reaching the point of discomfort (see Figure 1A), with the ankle plantar flexed to mitigate possible neural tension from limiting ROM. In the CR_dyna_ condition, the leg was held in the stretched position for 10 seconds followed immediately with a 5‐second ramped maximal isometric knee flexor contraction performed with the muscle at full stretch (ie, at point of discomfort [see Figure 1B]). Upon contraction cessation, the knee was immediately passively extended by the dynamometer (if participants were able) until reaching the new point of discomfort with the protocol repeated three further times giving a total duration of 60 seconds (ie, 4 × 10‐seconds stretches and 4 × 5‐seconds contractions). During the SRC_dyna_ condition, the static stretch phase was identical (see Figure 1C); however, immediately after the 10 seconds of stretching the knee was returned to the starting position (90° flexion, ie, “off stretch”) where the 5‐seconds ramped maximal isometric contraction was performed (see Figure 1D). The knee was extended again until reaching the point of discomfort with the protocol repeated three times giving a total duration of 60 seconds.

**Figure 2:**
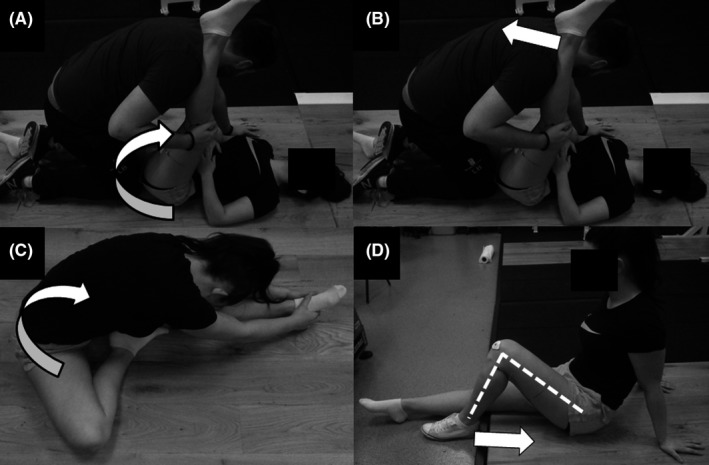
Participant positioning during field‐based contract‐relax (CR) and modified CR (stretch‐return‐contract [SRC]) stretching protocols. During the field‐based CR protocol, the straight leg raise (SLR) stretch was performed by a partner (A) before the contraction phase was performed at full stretch (B). During the field‐based SRC protocol, the participants performed a modified hurdler's stretch (C) before the contraction phase performed at 90° flexion (D)

During the field‐based conditions, the participants performed the stretches with (CR_field_) or without (SRC_field_) partner assistance. During the CR_field_ condition, the straight leg raise (SLR) technique was used to stretch the knee flexors. The participant was placed in a supine position with the partner straddling the participant's right leg to prevent anterior pelvic tilt, and with the participant's left heel on the partner's shoulder. The partner then flexed the participant's hip while maintaining knee extension until the point of discomfort (see Figure 2A). The leg was held in the stretched position for 10 seconds followed immediately with a 5‐second ramped maximal isometric contraction (partner's shoulder providing resistance [see Figure 2B]) performed with the muscle at full stretch (ie, point of discomfort). Upon contraction cessation, the hip was immediately flexed (if participants were able) until reaching the new point of discomfort with the protocol repeated three further times giving a total duration of 60 seconds (ie, 4 × 10‐second stretches and 4 × 5‐second contractions). During the SRC_field_ condition, the protocol was performed unaided with the participant seated and the static stretch phase completed using the modified hurdler's stretch (see Figure 2C). Immediately after the 10 seconds of stretching, the knee was flexed to 90° where a 5‐second ramped maximal isometric contraction was performed with the heel against a metal frame to provide resistance to the contraction (see Figure 2D), with the protocol repeated three times giving a total duration of 60 seconds. Two minutes later the participants repeated the passive and active trials in the dynamometer to determine the impact of the interventions on MTC mechanics.

### 2.3 Data analysis

All data were analyzed using SPSS statistical software (v.22; IBM) and are reported as mean (SD); Cohen's D was used to calculate effect size (ES). Normal distribution for pre‐ and post‐stretching data was assessed using Shapiro‐Wilk tests; no significant difference (*P* > .05) was detected in any measure indicating that all data sets were normally distributed. Separate two‐way repeated measures ANOVA’s were used to test for the effects of time (×2) and condition (×4) in (a) ROM, (b) peak passive joint moment (stretch tolerance), (c) area under the passive joint moment curve (elastic potential energy storage), (d) slope of the passive joint moment curve (MTC stiffness), (e) reflexive EMG, (f) peak EMG, and (g) peak isometric moment. Where significant effects were detected, post‐hoc t test analyses using Bonferroni correction were employed to determine the location of any differences. Pearson's product‐moment correlation coefficients (*r*) were computed to quantify the linear relationship between the changes in all variables in each condition. Statistical significance for all tests was accepted at *P* < .05.

### 2.4 Reliability

Test‐retest reliability was determined using pre‐intervention data across the four conditions by calculating intraclass correlation coefficients (ICC [3,1]) using a single rater, absolute‐agreement, two‐way mixed‐effects model and coefficients of variation (CV). No significant difference (*P* > .05) was detected in any measure (pre‐intervention) with excellent reliability reported for ROM (ICC = 0.93; CV = 2.1%), peak passive moment (ICC = 0.94; CV = 9.8%), area under the moment curve (ICC = 0.93; CV = 11.3%), the slope of the passive moment curve (ICC = 0.91; CV = 10.7%), and peak isometric moment (ICC = 0.97; CV = 6.0%).

### 2.5 Sample size

To ensure an adequate sample size was recruited for the study, effect sizes (Cohen's D) were calculated from mean changes in variables (ROM, passive moment, peak isometric moment) from previous studies employing similar methods.^15^, ^22^, ^23^, ^24^ To ensure statistical power for all variables, power analyses were conducted using the variable with the smallest effect size with the following parameters (power = 0.80, alpha = 0.05, effect size = 1.0, attrition = 20%). The analysis revealed that the initial sample size required to reach statistical power was 14, thus 18 participants were recruited to account for possible attrition or data loss. One participant failed to complete the four interventions; thus, statistical analyses were conducted on data sets for the 17 participants that completed the testing.

## 3 RESULTS

### 3.1 Range of motion and MTC stiffness

**Figure 3:**
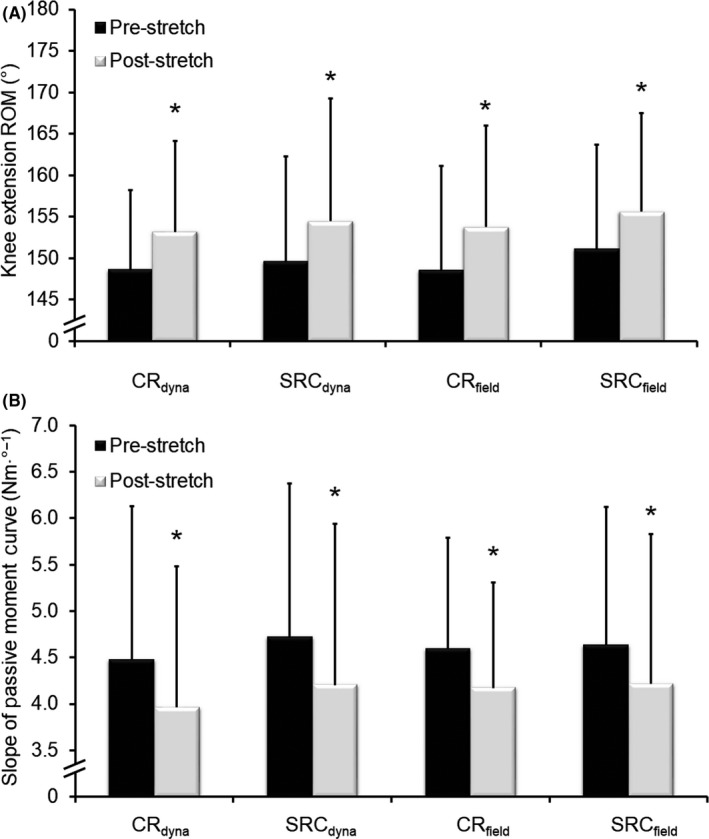
Mean (SD) knee extension range of motion (ROM) and muscle‐tendon complex (MTC) stiffness before and after dynamometry‐ (CR_dyna_ and SRC_dyna_) and field‐based (CR_field_ and SRC_field_) stretching protocols. Significant increases in ROM ([A] 4.6‐5.2°) and reductions in the slope of the passive moment curve ([B] 8.9%‐12.2%, indicative of MTC [joint] stiffness) were observed after all conditions. No difference was found between conditions in any measure. ^*^Significant to *P* < .05

Significant (*P* < .01) increases in knee extension ROM (see Figure 3A) were detected after CR_dyna_ (mean [SD] = 4.7 [5.6°], ES = 0.83), SRC_dyna_ (4.9 [5.8°], ES = 0.84), CR_field_ (5.2 [5.0°], ES = 1.04), and SRC_field_ (4.6 [3.8°], ES = 1.20) stretching conditions. No significant differences (*P* > .05) in ROM were detected between conditions. Significant (*P* < .01) decreases in the slope of the passive moment curve (indicative of knee flexor MTC stiffness) were detected after CR_dyna_ (11.4 [15.8%], ES = 0.72), SRC_dyna_ (12.2 [15.4%], ES = 0.79), CR_field_ (8.9 [9.3%], ES = 0.96), and SRC_field_ (8.9 [14.5%], ES = 0.62) stretching conditions (see Figure 3B), with no significant difference (*P* > .05) being detected between conditions. No significant correlations (*P* > .05) were found between changes in ROM and changes in MTC stiffness (*r* = .09‐.16) in any condition.

### 3.2 Elastic potential energy storage and peak passive joint moment (stretch tolerance)

**Figure 4:**
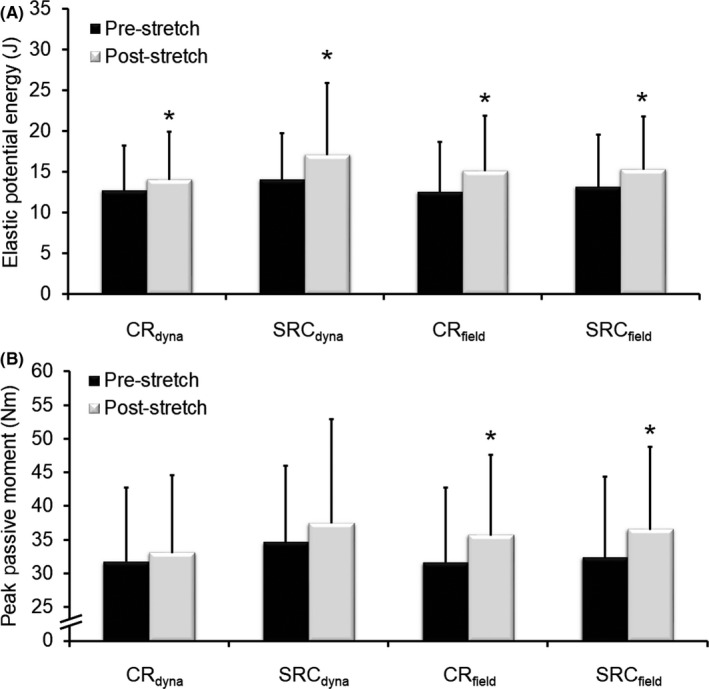
Mean (SD) elastic potential energy storage and peak passive joint moment (ie, stretch tolerance) before and after dynamometry‐ (CR_dyna_ and SRC_dyna_) and field‐based (CR_field_ and SRC_field_) stretching protocols. Significant increases in elastic potential energy storage (A) were observed in all conditions (12.0%‐23.6%). Significant increases in stretch tolerance (B) were observed after field‐ (14.3%‐14.8%) but not dynamometry‐based (4.6%‐6.6%) stretches. No differences were found between conditions in any measure. ^*^Significant to *P* < .05

**Figure 5:**
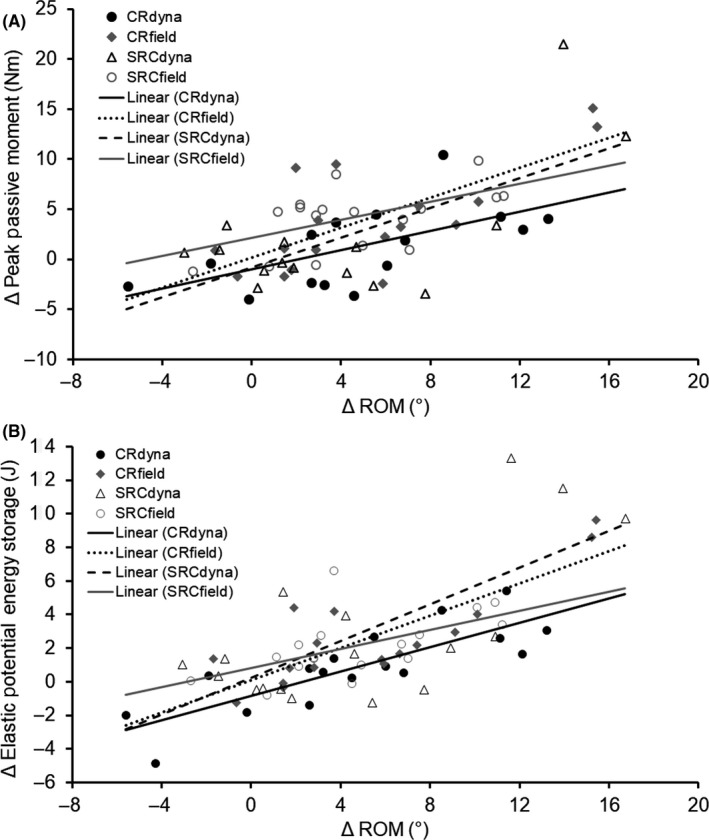
Correlations between changes (Δ) in knee extension range of motion (ROM) and changes in elastic potential energy storage and peak passive joint moment (ie, stretch tolerance) after dynamometry‐ (CR_dyna_ and SRC_dyna_) and field‐based (CR_field_ and SRC_field_) stretching protocols. Significant correlations (*P* < .05) were detected between changes in ROM and changes in elastic potential energy storage ([A] *r* = .58‐.84) and changes in stretch tolerance ([B] *r* = .55‐.73) and in all conditions

Significant increases (*P* < .01) in elastic potential energy storage during stretch were found after CR_dyna_ (mean [SD] = 12.0 [20.1%], ES = 0.60), SRC_dyna_ (15.7 [29.8%], ES = 0.53), CR_field_ (23.6 [26.3%], ES = 0.89), and SRC_field_ (21.4 [20.0%], ES = 1.13) stretching conditions (see Figure 4A). No significant difference (*P* > .05) in elastic potential energy storage was detected between conditions. Significant increases (*P* < .01) in peak passive moment (stretch tolerance) were detected after CR_field_ (mean [SD] = 14.3 [16.8%], ES = 0.85) and SRC_field_ (14.8 [11.4%], ES = 1.29) stretching conditions, but not after CR_dyna_ (mean [SD] = 4.6 [16.3%], ES = 0.28) or SRC_dyna_ (6.6 [18.5%], ES = 0.36) stretching conditions (see Figure 4B). Nonetheless, no significant difference (*P* > .05) in stretch tolerance was detected between conditions. Significant positive correlations (*P* < .05) were detected between absolute changes in ROM and absolute changes in stretch tolerance (*r* = .55‐.73) and elastic potential energy storage (*r* = .58‐.84) in all conditions (see Figure 5).

### 3.3 Isometric knee flexor moment and EMG

During the MVC trials, no significant difference (*P* > .05) in maximal isometric knee flexor moment was detected in any condition (mean range = −3.3 to −5.6%MVC) or between conditions during pre‐ (mean range = 105.4 to 109.0 Nm) or post‐intervention (mean range = 98.5 to 106.1 Nm) trials. No significant difference in peak EMG activity was detected in any condition (mean range = −6.7 to 11.8%MVC) or between conditions during pre‐ (mean range = 102.9 to 108.6%MVC) or post‐intervention (mean range = 96.8% to 104.4%MVC) trials. During the passive ROM trials, minimal activation occurred with no significant difference in reflexive EMG activity detected in any condition (mean range = −1.2 to 0.7%MVC) or between conditions during pre‐ (mean range = 1.8 to 3.7%MVC) or post‐intervention (mean range = 1.6 to 4.4%MVC) trials.

## 4 DISCUSSION

Similar increases in dorsiflexion ROM (ie, plantar flexor flexibility), decreases in both muscle and tendon stiffness, as well as no changes in neuromuscular activity (during stretch) have previously been reported following acute bouts of CR and modified CR (SRC) stretching when performed using an isokinetic dynamometer.^16^ However, it is not known whether the SRC method is effective when performed by an individual without partner or dynamometer assistance or in muscle groups other than the plantar flexors. Thus, the ecological validity of the method remained unknown. In the present study, similar increases in knee flexor ROM (ie, hamstring extensibility) were evoked by classical CR and SRC stretching, that is, there was no difference when performing the muscle contraction on stretch vs off stretch. This finding is consistent with previous findings in the plantar flexors when the stretching was performed in an isokinetic dynamometer^16^ and confirmed the efficacy of the SRC technique in other muscle groups. Although PNF‐based stretching techniques (eg, contract‐relax [CR]) are commonly reported to generate greater acute increases in ROM than other stretching methods,^8^, ^9^, ^15^ these techniques have several disadvantages that may restrict their common use in clinical and athletic environments. Performing intense muscle contractions at long muscle lengths, that is, at full stretch, can be painful and induce greater symptoms of muscle tissue damage.^12^, ^13^ Furthermore, populations with hypertonic symptoms arising from spasticity or contracture,^14^ performing this technique might be problematic as individuals lose full ROM and are, therefore, unable to fully stretch the muscle. Therefore, as the muscle contraction phase was performed “off stretch” when using the SRC technique, the data indicate that SRC stretching is an equally effective but potentially more practical stretching technique than classical PNF techniques to assist with clinical exercise prescription in a range of conditions currently unable to effectively use current PNF stretching techniques.

While bands or a towel can be used by an individual to create resistance during the isometric contraction phase of classical PNF in some muscle groups (eg, hamstrings), the primary practical limitation of classical CR stretching is often the need for a partner or clinician to hold the limb during the intense muscular contractions.^8^, ^9^ This limitation can prevent patients (at home following clinical discharge) and others (eg, athletes) from implementing these strategies on their own. The need for partner or clinician assistance during PNF‐based stretching was a particularly important consideration in the present study as it limits the practicality, and thus, use of PNF stretching despite it being regularly reported to induce the greatest mean increases in ROM.^8^, ^9^, ^10^ Thus, comparing the effects of the two stretch techniques (ie, CR vs SRC) on knee extension ROM in both laboratory‐ and field‐based environments was an important aim. In agreement with our hypothesis, consistent ROM increases were demonstrated in both environments, confirming the ecological validity of the SRC technique to be used in an applied setting. As the field‐based SRC technique was completed without partner assistance and with the muscle contractions performed at shorter muscle lengths, the modified technique can be considered to be a more practical, yet equally effective, stretching paradigm. However, it was decided in the present study to confirm the ecological validity of the technique in a healthy population, prior to use in clinical populations such as those with diabetes,^2^, ^3^ cerebral palsy,^4^ stroke,^5^ arthritis,^7^ or in elderly individuals.^6^ Nonetheless, as similar improvements in ROM were achieved as with CR stretching (ie, current clinical “gold standard”) but without the significant practical limitations that restrict the use of CR stretching in clinical and other (eg, athletic) environments, these findings likely have important implications for clinical exercise prescription in populations where ROM is often compromised.

Of interest is that similar reductions in the slope of the passive joint moment‐angle relation (~9%‐12%) were found in all conditions in the present study, indicating a reduction in MTC [joint] stiffness. This finding is consistent with previous studies imposing CR stretching at the knee^17^ as well as both CR and SRC at the ankle.^16^ However, no significant correlation was found between increases in ROM and the reduction in MTC stiffness in any condition, indicating that other mechanisms might more prominently underpin the acute changes in ROM. Additionally, no substantial EMG activity (ie, observed activity was <5%MVC) was observed in any condition during the passive ROM trials, consistent with a recent study examining the effects of CR and SRC in the plantar flexors.^16^ As no substantial activation of the α‐motoneuron pool appears to have occurred in any condition, alterations in autogenic inhibition are also not likely to be an important mechanism underpinning the increases in ROM, which is also consistent with the conclusions presented in reviews on PNF‐based stretching techniques.^9^, ^11^ However, a limitation of the present study was that subject positioning in the modified SRC condition prevented EMG activity recording during the stretches and therefore, comparison between stretches, thus further analyses during these stretches are required to confirm this hypothesis. Nonetheless, a neurological contribution is at least partly supported by the increase in peak passive joint moment (ie, stretch tolerance) after field‐based stretches, and significant correlations (*r* = .55‐.73; *P* < .05) between the changes in peak passive moment and maximum ROM in all conditions. These data are consistent with previous studies examining responses to knee^17^, ^18^ and plantar flexor^15^, ^16^, ^25^ muscle stretching and are indicative that increased stretch tolerance is likely an important mechanism influencing ROM after both CR and SRC stretching. Nonetheless, the specific neuromuscular pathways influencing stretch tolerance remain to be established.

## 5 PERSPECTIVES

The present study is the first to examine the acute effects of performing the muscle contraction phase of CR stretching in shortened muscle lengths (ie, “off stretch”) without partner or dynamometer assistance. Comparable increases in knee flexor ROM, reductions in stiffness and increases in stretch tolerance and elastic potential energy storage were observed after both CR and SRC stretching when performed in both laboratory‐ (dynamometer) and field‐based environments. The ability of the SRC technique to generate similar improvements in ROM to classical CR (ie, PNF) stretching has important practical implications since performing the contractions “off stretch” is painless. Furthermore, the removal of the need for partner/clinician assistance also lends itself to use in applied athletic, clinical, and outpatient environments. Therefore, while PNF‐based stretching techniques such as the CR method is often considered the “gold standard” method for improving ROM, the SRC stretching technique may offer a more practical yet equally effective stretching model. Based on the results of the present proof of concept study in a healthy population, tests in clinical and other (eg, athletic) populations are warranted. Furthermore, the effects of prolonged SRC training on chronic ROM and muscle‐tendon adaptations should also be tested.
